# The impact of bacterial and viral diseases on dromedary camel (*Camelus dromedarius*) welfare: a comprehensive review

**DOI:** 10.3389/fvets.2026.1795334

**Published:** 2026-05-29

**Authors:** Syed Faizan Ali Shah, Mohamed Tharwat, Abdul Rehman, Fahad A. Alshanbari

**Affiliations:** 1 Department of Veterinary Medicine, University of Veterinary and Animal Sciences, Lahore, Pakistan; 2 Department of Clinical Sciences, College of Veterinary Medicine, Qassim University, Buraidah, Saudi Arabia; 3 Department of Epidemiology and Public Health, University of Veterinary and Animal Sciences, Lahore, Pakistan; 4 Department of Medical Biosciences, College of Veterinary Medicine, Qassim University, Buraidah, Saudi Arabia

**Keywords:** animal welfare, dromedary camel, infectious diseases, one health–one welfare, zoonoses

## Abstract

Dromedary camels (*Camelus dromedarius*) are vital to food security, livelihoods and cultural identity in arid and semi-arid regions of Africa, the Middle East and South Asia, with global populations exceeding 35–40 million and continuing to rise as climate change favors livestock adapted to harsh environments. Despite their expanding roles in meat and milk production, transport, tourism and racing, camel health and welfare remain comparatively underexplored, particularly regarding infectious diseases. This review synthesizes current knowledge on major bacterial infections, including brucellosis, tuberculosis, mastitis, suppurative infections, salmonellosis, and clostridial diseases, as well as important viral diseases such as Middle East respiratory syndrome coronavirus, camel pox, Rift Valley fever, rabies, contagious ecthyma, and peste des petits ruminants, with a focus on their implications for animal welfare. These diseases commonly lead to acute and chronic pain, fever, respiratory compromise, reproductive failure, debilitation, and mortality, and are associated with physiological stress responses such as activation of the hypothalamic–pituitary–adrenal axis and increased acute-phase proteins, alongside behavioral changes including lethargy, reduced grooming, altered social interactions, and impaired maternal and working behaviors. Using the Five Freedoms and the Five Domains Model frameworks, this review demonstrates how infectious diseases undermine multiple dimensions of camel welfare, including nutrition, physical comfort, health, behavior, and mental state. Beyond individual animals, camel diseases have significant socioeconomic and ethical implications. Several pathogens are zoonotic, posing risks to pastoralists, veterinarians, and other stakeholders, and influencing trade regulations, disease control strategies, and culling practices, which may further exacerbate welfare compromise. Key knowledge gaps remain, including the lack of validated camel-specific welfare assessment tools, limited longitudinal data on welfare trajectories during disease outbreaks, and insufficient integration of welfare indicators into surveillance and control programs. The review highlights priority research and policy needs, including the development of field-adapted welfare scoring systems, evaluation of welfare impacts of interventions such as vaccination and movement restrictions, and stronger integration of animal welfare into One Health and One Welfare frameworks to support sustainable camel production and resilient dryland livelihoods.

## 1 Introduction

### 1.1 Importance of dromedary camels

Dromedary camels dominate the global camel population, accounting for roughly 95% of large camelids (1). Recent FAO-linked analyses estimate that there were about 35.5 million camels in 2018, with current figures likely exceeding 40 million as camelid numbers continue to rise (2). Most dromedaries are kept in the Sahelian and East African belt (Somalia, Sudan, Ethiopia, Kenya, Chad, Niger, Mali and Mauritania), the Arabian Peninsula (Saudi Arabia, United Arab Emirates, Oman, Qatar and Yemen), and parts of South Asia (Pakistan and India), with smaller populations in Central Asia and Australia (3).

Economically, camels provide meat, milk, hides, hair and draught power (4). In some sub-Saharan African countries, they contribute around 8% of total milk production and represent an important source of animal protein where other livestock struggle under harsh climatic conditions (5). Camel milk and meat are increasingly commercialized, with growing urban demand and niche markets in Europe and North America (6). Culturally, camels hold symbolic value in many pastoral societies, playing roles in social status, dowry systems, festivals and racing.

Ecologically, dromedaries are well adapted to arid and semi-arid ecosystems through their ability to browse shrubs, utilize saline water and cope with extreme temperatures. As climate change intensifies drought and heat stress, camels are being reconsidered as climate-resilient livestock, and camel dairies are expanding in North Africa, the Arabian Peninsula and South Asia (7, 8). However, rapid intensification and commercialization have not been matched by equivalent investments in disease control, welfare assessment and veterinary infrastructure (9).

### 1.2 Understanding animal welfare in camels

**Figure 1:**
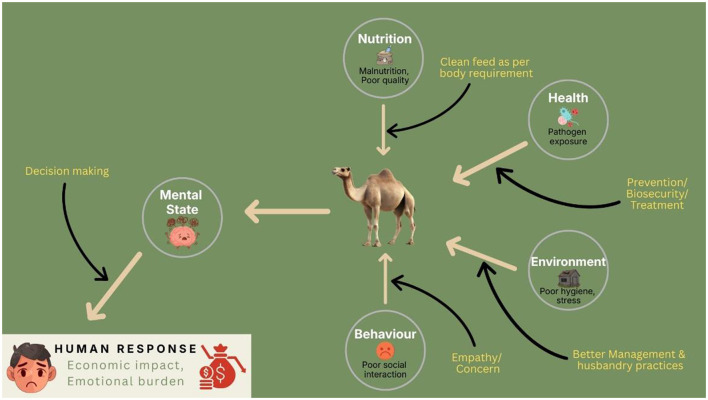
Conceptual framework of the five domains model for assessing camel welfare. The model integrates five interconnected domains—nutrition, environment, health, behavior and mental state—to evaluate the overall welfare status of camels.

Animal welfare is commonly conceptualized using the “Five Freedoms” (freedom from hunger and thirst; discomfort; pain, injury or disease; fear and distress; and freedom to express normal behavior) and more recently the Five Domains Model, which evaluates nutrition, environment, health, behavioral interactions and mental state (10, 11). Each domain influences and interacts with others: nutrition affects health and behavior; environmental quality impacts health and mental state; and behavioral and mental conditions are influenced by human empathy, management, and decision-making (Figure 1). The framework also highlights the human response, emphasizing the economic and emotional consequences of welfare-related issues and the role of effective management, biosecurity, and empathetic husbandry in improving camel wellbeing. The Five Domains Model is now widely used for systematic, structured, and holistic welfare assessment, including in camels (12, 13).

Camel-specific welfare challenges include rough handling during loading, racing and transport; inadequate access to water and balanced feed in extensive systems; poor shade and heat mitigation; and limited routine veterinary care. Recent work has begun to develop structured welfare assessment protocols for dromedaries, adapting the Five Domains to camel behavior and husbandry contexts (14–16). Diseases intersect with each of these domains: they reduce feed intake and body condition (nutrition), induce fever and respiratory distress (health and environment), alter social and grooming behavior (behavioral interactions) and generate negative affective states such as pain and distress (mental state) (17).

### 1.3 Rationale and objectives

A recent report demonstrated that parasitic diseases severely compromise camel welfare, while revealing gaps in species-specific assessment and surveillance systems. It highlights the links between camel welfare, human health via zoonotic parasites, and the socio-economic stability of pastoral communities (18).

Compared with cattle, small ruminants and poultry, integrative reviews explicitly linking infectious disease to camel welfare are rare. Most literature has traditionally emphasized economic losses, zoonotic risks or basic epidemiology (19–21). Yet welfare science offers powerful tools for evaluating the full impact of disease and for designing more humane control strategies.

Electronic databases including PubMed, Web of Science, Scopus, and Google Scholar were searched using combinations of relevant keywords such as “*Camelus dromedarius”*, “zoonotic implications of camels”, “camel herd health”, “economic impacts”, “One Health” and “camel welfare”. Although this was not a formal systematic review, the search was designed to capture recent and relevant evidence linking camel diseases with welfare and socioeconomic outcomes, providing a broad and informed basis for the present study. This review therefore aims to: (i) summarize major bacterial and viral diseases of dromedary camels with relevance to welfare, (ii) evaluate their direct and indirect effects on physical, physiological and behavioral welfare indicators within the Five Domains framework and, (iii) identify knowledge gaps and propose welfare-oriented disease management and policy strategies within a One Health/One Welfare perspective.

## 2 Overview of infectious diseases in dromedary camels

### 2.1 Epidemiological context

Dromedaries are affected by a wide spectrum of infectious agents including bacteria (e.g., *Brucella* spp., *Mycobacterium bovis, Staphylococcus* spp., *Corynebacterium pseudotuberculosis, Escherichia coli*), viruses (camel pox virus, Middle East respiratory syndrome coronavirus—MERS-CoV, Rift Valley fever virus—RVFV, rotaviruses, adenoviruses), parasites and mycotic agents (22, 23). Many of these diseases are transboundary, and are facilitated by cross-border pastoralism, camel trading networks, racing events and religious pilgrimages, which increase opportunities for pathogen transmission (24, 25).

Husbandry factors strongly influence disease occurrence and its spread. Nomadic and transhumant systems involve long-distance movements, shared water points and mixed-species grazing with cattle, sheep and goats, all of which increase exposure to contaminated environments and vectors (26, 27). Limited access to vaccination, sparse veterinary infrastructure and under-reporting further hinder effective disease control and surveillance (20, 28). Climate-driven changes in vector ecology, such as mosquito populations relevant to RVFV, may also alter disease risk profiles. These factors increase not only infection pressure but also prolonged and unmanaged suffering.

Beyond the biological characteristics of each pathogen, management and environmental factors are central drivers of infection pressure in camel herds. Key husbandry-related risk factors for major bacterial and viral diseases are summarized in Table 1.

**Table 1:** Husbandry and management risk factors for bacterial and viral diseases in dromedary camels.

Sr No	Husbandry factors	Specific risk factor	Main diseases	Welfare consequences	References
1	Housing & environment	Overcrowded night enclosures	Mastitis, suppurative infections, camel pox	Increased aggression, injuries, difficulty in resting	(29, 30)
2	Water access	Shared water points with multiple species	Brucellosis, TB, RVF	Prolonged waiting, social tension, heat stress	(31–33)
3	Reproductive management	Unhygienic assistance at calving	Metritis, reproductive tract infections	Pain, prolonged recovery, poor maternal care	(33, 34)
4	Movement & trade	Long-distance trekking to markets	Respiratory viruses, MERS-CoV, pox	Exhaustion, dehydration, transport-related injuries	(35–37)
5	Vector exposure	Uncontrolled mosquito and biting-fly populations	RVF, other arboviruses	Persistent irritation, disturbed rest, anaemia	(32, 38)
6	Human–animal interface	Raw milk and close household contact	Brucellosis, TB, MERS-CoV (for humans)	Fear-based reactions to diseased camels, rough handling	(23, 33)
7	Veterinary access	Limited veterinary visits	All infectious diseases	Delayed treatment, prolonged pain and suffering	(16)

### 2.2 Classification of diseases

For welfare-oriented discussion, diseases can be broadly grouped into; Bacterial diseases including brucellosis, tuberculosis, suppurative conditions (abscesses, wound infections, ulcerative lymphangitis), mastitis and reproductive tract infections. Many of these have zoonotic potential (20, 39). Viral diseases including MERS-CoV, camel pox, RVFV and a range of emerging or less-studied viruses (parapoxvirus, rotavirus, adenovirus, enteric coronaviruses) (22, 40). Both groups cause acute and chronic welfare challenges. Acute infections may lead to sudden fever, pain and mortality, whereas chronic diseases such as tuberculosis, brucellosis-related arthritis or unresolved skin lesions, abortion and infertility cause prolonged suffering and progressive decline.

## 3 Major bacterial diseases affecting camel welfare

### 3.1 Brucellosis

Brucellosis is endemic in many camel-keeping countries in Africa, the Middle East and South Asia, where camels often co-graze with infected cattle, sheep and goats (25, 41, 42). The main camel-associated species are *B. melitensis* and *B. abortus*, which are important zoonotic pathogens. Infection occurs mainly through ingestion or contacts with aborted material and contaminated milk and sometimes through coitus (43, 44). Pathogenesis involves colonization of the reproductive tract and reticuloendothelial system, leading to late-term abortions, stillbirths, retained placenta, infertility, orchitis and epididymitis. Joint involvement causes arthritis and hygromas. These manifestations translate into severe welfare compromise: recurrent abortions, chronic fever, joint swelling and pain reduce mobility, body condition and maternal behavior (45).

Brucellosis is also a major public health threat to pastoralists, abattoir workers and veterinarians through consumption of raw milk or occupational exposure (33, 46). Human cases are often linked to direct contact with infected animals or their products, with symptoms ranging from fever and joint pain to chronic illness. Low awareness, lack of compensation, and fear of infection can lead to premature culling or rough handling of camels, creating ethical and livelihood tensions (43).

### 3.2 Camel tuberculosis

Tuberculosis (TB) in camels is mainly caused by members of the *Mycobacterium tuberculosis* complex such as *M. bovis, M. caprae*, and atypical *mycobacteria* (47–49). Infection is usually chronic, with non-specific signs, and is frequently under-diagnosed in the field due to limited use of tuberculin testing, culture or molecular diagnostics (50, 51).

Clinically affected animals may show progressive weight loss, chronic respiratory signs (coughing, dyspnea), weakness and reduced productivity. Lesions in the lungs and lymph nodes compromise respiratory function and general health, leading to prolonged suffering (50). From a welfare standpoint, TB exemplifies a disease where low-grade but persistent pain, dyspnea and malaise can last months or years. Additionally, TB is zoonotic; close contact and consumption of raw camel milk pose risks to humans, particularly in communities where pasteurization is uncommon (52). Test-and-slaughter strategies raise ethical concerns in camels because weak welfare oversight, limited species-specific legislation, and the high social and economic value of individual animals make large-scale slaughtering difficult to justify without clear evidence, transparent decision-making, and strong measures to minimize suffering and protect pastoral livelihoods (53).

### 3.3 Suppurative conditions

Suppurative diseases are common in working and free-ranging camels due to minor injuries, harness lesions, ticks and contaminated husbandry environments. *Corynebacterium pseudotuberculosis* is associated with caseous lymphadenitis and ulcerative lymphangitis, whereas *Staphylococcus aureus* and other staphylococci are frequent causes of abscesses and wound infections (54, 55), whereas *Staphylococcus lugdunensis, Staphylococcus aureus*, and *Escherichia coli*, are commonly associated with pyelonephritis, renal abscessations and urinary tract infections (56, 57).

These infections manifest as painful swellings, draining tracts and lameness. In pack or riding camels, abscesses at the withers or girth region directly impair working ability and cause intense local pain under load. Chronic suppurative lesions may also predispose to systemic illness and stigma within communities who perceive visibly diseased animals as unclean or unfit for ceremonial or market use (58).

### 3.4 Mastitis and reproductive tract infections

Mastitis in dairy camels is increasingly reported as milking intensifies and peri-urban camel dairies expand. *Staphylococcus aureus, Streptococcus spp*. and coliforms such as *E. coli* are common etiological agents (59, 60). Clinical mastitis manifests as udder swelling, heat, pain, abnormal milk and systemic signs. Subclinical mastitis may go unnoticed but still cause discomfort and reduced yield. Welfare consequences include nursing difficulties, maternal agitation, reluctance to be milked and altered maternal–calf interactions (61, 62).

Metritis, vaginitis, and endometritis are common reproductive tract infections in camels, often following dystocia, retained placenta, or unhygienic obstetrical interventions (63, 64). *E. coli, Staphylococcus* spp., and Streptococcus spp. are commonly recovered from infected reproductive tracts in camels. Such infections can result in chronic pain, malodorous discharges, infertility, and repeated unsuccessful breeding attempts, frequently culminating in culling and raising serious welfare concerns (65). Among these, uterine lesions are the most frequently observed, often linked to heavy bacterial loads and significant reductions in reproductive performance (66).

### 3.5 Salmonellosis

Salmonellosis in dromedary camels is most commonly associated with *Salmonella enterica* serovars and tends to occur intermittently, particularly in situations involving stress such as transportation, high stocking density, inadequate hygiene, or concurrent disease. Reported prevalence in camel populations is generally low to moderate (approximately 5%−10%), with many infections remaining subclinical; nonetheless, clinically apparent cases with systemic involvement have been documented (67, 68). Evidence from food safety studies indicates that camel-derived products may serve as sources of infection, with investigations in Egypt identifying *Salmonella* contamination in about 10% of raw camel milk samples, including isolates exhibiting multidrug resistance (69). The clinical expression of salmonellosis in camels varies widely, ranging from asymptomatic carriage to severe enteric disease, septicemia, and reproductive losses. Symptomatic animals commonly exhibit diarrhea, fever, dehydration, and generalized weakness, all of which contribute to marked discomfort and physiological stress. Young camels are particularly vulnerable, as rapid fluid and electrolyte losses can severely compromise welfare and significantly elevate mortality risk. Importantly, subclinically infected camels also represent a welfare and public health concern, as persistent carriers can sustain transmission within herds and increase the risk of food-borne zoonotic exposure (70).

### 3.6 Anthrax

Anthrax is widely recognized by the World Organization for Animal Health (WOAH) and regional assessments as an important disease affecting camels within endemic zones, characterized by high fatality rates and substantial zoonotic risk associated with infected carcasses and animal products (24). The disease is caused by *Bacillus anthracis* and, although sporadic, can result in acute outbreaks among camels in affected regions (71). Clinical onset is typically rapid, with affected animals exhibiting sudden death or signs of severe systemic illness, including high fever, respiratory compromise, edema, and hemorrhagic discharges. Owing to its peracute course, anthrax results in profound but often brief welfare impairment; however, animals that survive long enough to develop clinical signs may endure extreme pain, distress, and physiological dysfunction. In addition to the direct effects of the disease, control interventions—such as emergency slaughter, strict carcass disposal procedures, and movement restrictions—can indirectly compromise the welfare of unaffected animals by limiting access to grazing areas and water sources. Furthermore, heightened concern over zoonotic transmission may lead to neglect, abandonment, or rough handling of suspected cases, raising significant ethical and welfare challenges (72, 73).

### 3.7 Pasteurellosis

Pasteurellosis, most commonly associated with *Pasteurella multocida* and *Mannheimia haemolytica* (formerly classified within the *Pasteurella* genus), represents a significant respiratory disease in camels, particularly under conditions of stress such as transportation, sudden climatic changes, or poor nutrition (73). Affected camels typically develop fever, nasal discharge, coughing, labored breathing, and marked lethargy. From a welfare standpoint, impairment of respiratory function is of major concern, as breathing difficulty is commonly accompanied by distress, exhaustion, and reduced tolerance for physical activity or work (74). In the absence of timely and appropriate treatment, acute infections may evolve into chronic pneumonia, leading to extended periods of discomfort, progressive weight loss, and diminished productivity. Early recognition of clinical signs and provision of supportive veterinary care are therefore critical for minimizing welfare compromise in affected camels (75, 76).

### 3.8 Clostridial diseases

Clostridial diseases in camels, such as enterotoxemia, tetanus, and malignant edema, arise from infections with toxin-producing *Clostridium* species (77). These conditions are typically characterized by rapid onset and high fatality rates. Enterotoxemia is associated with severe abdominal discomfort, diarrhea, neurological disturbances, and sudden death, whereas tetanus manifests as persistent muscle stiffness, heightened sensitivity to external stimuli, and marked impairment of feeding and locomotion. From a welfare perspective, clostridial infections represent some of the most severe disease-related challenges in camels, as affected animals endure extreme pain, psychological distress, and significant functional limitations. Contributing risk factors include inadequate wound hygiene, reliance on traditional management practices, and insufficient vaccination coverage. Consequently, routine preventive immunization and appropriate wound care are critical interventions for reducing disease occurrence and safeguarding camel welfare (78–80).

### 3.9 Q fever

Q fever is an increasingly recognized zoonotic infection in dromedary camels, caused by *Coxiella burnetii* (81). In most cases, infection remains clinically inapparent; however, adverse reproductive outcomes, including abortion, stillbirth, and the birth of weak offspring, have been documented. Despite the absence or mildness of overt clinical signs, the welfare consequences can be considerable, particularly for breeding females. Cross-sectional investigations conducted in Jordan and Kenya have identified associations between camel infection and factors such as heavy tick infestation, advanced age, and co-rearing with large goat populations (82, 83). Reproductive failure linked to Q fever may result in repeated breeding attempts, increased nutritional demands, and intensified management practices involving more frequent handling or confinement, all of which can further compromise welfare. Moreover, the zoonotic risk posed by *C. burnetii* may influence human responses to infected herds, leading to herd isolation, reduced husbandry attention, or early culling decisions (84). Owing to its largely silent transmission dynamics, Q fever represents a concealed yet significant threat to welfare within camel populations.

### 3.10 Paratuberculosis (Johne's disease)

Paratuberculosis, also known as Johne's disease, is a chronic, progressive granulomatous enteritis of ruminants caused by *Mycobacterium avium* subspecies *paratuberculosis* (MAP). Although historically under-studied in camelids, evidence increasingly demonstrates that dromedary camels (*Camelus dromedarius*) are susceptible to MAP infection with clinical and subclinical presentations documented across Africa, the Arabian Peninsula, and South Asia (85). In camels, clinical disease is characterized by chronic or intermittent diarrhea, severe weight loss (emaciation), reduced milk yield, dehydration, submandibular edema, and progressive decline in body condition, all of which directly undermine individual welfare and productivity (86). Infected animals may also display hematologic and biochemical abnormalities reflecting systemic deterioration (e.g., hypoproteinemia, anemia, and electrolyte imbalances), further indicating compromised health status. MAP infection in camels often remains subclinical for extended periods, facilitating environmental shedding and within-herd transmission while delaying diagnosis and control efforts (87). The prolonged incubation period of Johne's disease, combined with the limited sensitivity of diagnostic tests during early stages of infection, intensifies its welfare consequences, as camels are often identified only after advanced intestinal damage and pronounced clinical deterioration have occurred (88). Reports of high seroprevalence in certain regions suggest that infection may be widely distributed within camel populations, leading to negative effects on milk yield, body condition, and overall welfare. In light of these substantial welfare and productivity losses, Johne's disease is emerging as an important challenge for camel health management in both pastoral and intensive production systems (89).

Table 2 summarizes the principal bacterial diseases affecting dromedary camels, outlining their key clinical manifestations, modes of transmission, associated welfare impacts, and zoonotic potential. This comparative overview facilitates rapid assessment of disease severity and implications, thereby supporting evidence-based decision-making for targeted health interventions and informed policy development in camel management systems.

**Table 2:** Major bacterial diseases affecting dromedary camels: clinical features, welfare impacts, and zoonotic relevance.

Disease	Causative agent(s)	Key clinical manifestations	Primary welfare impacts	Chronicity	Zoonotic relevance
Brucellosis	*Brucella melitensis, B. abortus*	Abortion, infertility, arthritis, orchitis	Chronic pain, reproductive distress, impaired maternal behavior	Chronic	High
Tuberculosis	*Mycobacterium bovis* complex	Weight loss, chronic cough, dyspnea	Prolonged respiratory distress, malaise	Chronic	High
Mastitis	*Staphylococcus aureus, Streptococcus* spp., *E. coli*	Udder pain, abnormal milk	Pain, nursing difficulty, maternal stress	Acute–chronic	Moderate
Suppurative infections	*Corynebacterium pseudotuberculosis, Staphylococcus* spp.	Abscesses, lameness	Localized pain, impaired locomotion	Chronic	Low–moderate
Salmonellosis	*Salmonella enterica*	Diarrhea, fever, abortion	Dehydration, weakness, distress	Acute	High
Anthrax	*Bacillus anthracis*	Sudden death, hemorrhage	Severe acute distress	Peracute	Very high
Pasteurellosis	*Pasteurella multocida, Mannheimia haemolytica*	Pneumonia, dyspnea	Respiratory distress, fatigue	Acute–chronic	Low
Clostridial diseases	*Clostridium* spp.	Enterotoxemia, tetanus	Severe pain, neurological distress	Acute	Low
Q fever	*Coxiella burnetii*	Abortion, subclinical infection	Reproductive loss, chronic stress	Chronic	High
Paratuberculosis	*Mycobacterium avium* subspecies *paratuberculosis*	Diarrhea, emaciation	Progressive debilitation	Chronic	Low–possible

## 4 Major viral diseases affecting camel welfare

### 4.1 Middle East Respiratory Syndrome (MERS)

Dromedary camels are widely recognized as the primary reservoir for Middle East Respiratory Syndrome coronavirus (MERS-CoV), supported by extensive serological and virological evidence from numerous countries across the Middle East and Africa (37, 90). In camels, infection is usually mild or subclinical, characterized by transient nasal discharge and mild respiratory signs. Direct clinical welfare impacts at the individual level may therefore be limited in many cases.

However, the indirect welfare effects are substantial. Concern over zoonotic transmission has led to movement restrictions, trade bans, reduced demand for camel products and, in some situations, culling or stigmatization of camels (35, 91). Owners may resort to abrupt sale or neglect of animals perceived as risky to human health. These human-driven responses, policy decisions, and altered human–animal relationships illustrate how a predominantly subclinical infection in camels can precipitate major welfare and socioeconomic consequences (91, 92).

### 4.2 Camel pox

Camel pox is one of the most clinically significant viral diseases in dromedaries (93). It is endemic in many camel-keeping regions and causes outbreaks with variable morbidity and mortality, particularly in young animals (94). The disease presents with fever, depression and characteristic skin lesions—papules, vesicles and nodules—on the head, neck, limbs, lips, nostrils and udder. Lesions can become ulcerated and secondarily infected by bacteria (95, 96). The welfare impact is high: affected animals experience widespread cutaneous pain and pruritus, reluctance to move, difficulty feeding if oral lesions are present and potential impediments to nursing in lactating females. Severe cases may result in emaciation and secondary pneumonia. In working camels, visible lesions may also lead to social stigma and exclusion from tourism or racing, indirectly affecting how owners value and care for them (97).

### 4.3 Rift Valley Fever (RVF)

Rift Valley Fever (RVF) is caused by an arbovirus transmitted primarily by mosquitoes and affects a broad range of ruminants, including camels. Although literature on camels is less extensive than for sheep and cattle, outbreaks in East Africa and the Arabian Peninsula have documented abortions, high neonatal mortality, fever, jaundice and hepatic necrosis in camels ranging from 9 to over 30% in affected regions (98–100). The welfare dimension is profound: acute systemic illness with high fever, intense malaise and rapid death in young animals represents severe negative affective states. Abortion storms cause suffering in pregnant females and loss of maternal investment (101). Additionally, RVF virus is a serious zoonosis causing severe influenza-like illness, hemorrhagic fever and encephalitis in humans; veterinarians and herders involved in handling aborted materials are particularly at risk. The dual impact on animal and human health exemplifies One Health and One Welfare interdependencies (102, 103).

### 4.4 Rabies

Camels are fully susceptible to rabies; cases are reported from most camel-raising countries (UAE, Oman, Saudi Arabia, Morocco, Niger, Nigeria, Sudan, India, Iran, China, etc.) but remain sporadic relative to dog and wildlife cases (104). Rabies is a fatal zoonotic viral disease caused by lyssaviruses and, although rare in dromedary camels, has been reported sporadically in regions where rabies is endemic in domestic dogs and wildlife. Camels typically acquire infection through bite wounds, and clinical disease is characterized by progressive neurological dysfunction. Affected camels may exhibit abnormal aggression, hyperexcitability, excessive salivation, incoordination, and paralysis. From a welfare standpoint, rabies represents one of the most severe infectious threats to camels, as it induces intense neurological dysfunction, heightened fear responses, and is invariably fatal once clinical signs emerge due to the absence of effective treatment (105). In Oman, camels exposed through bites from suspected rabid animals are commonly quarantined and treated as rabies suspects, with humane euthanasia frequently implemented after laboratory confirmation in order to address both animal welfare considerations and the substantial zoonotic risk (106). The zoonotic risk associated with rabid camels also alters human–animal interactions, frequently leading to abrupt isolation or killing of suspect animals, sometimes under suboptimal welfare conditions. Preventive vaccination of reservoir species and improved bite-wound management are therefore important not only for public health but also for preventing extreme welfare outcomes in camels (107).

### 4.5 Camel Contagious Ecthyma

Camel Contagious Ecthyma (CCE) is a highly contagious parapoxvirus infection producing papules, vesicles, pustules and thick crusts on the lips, nostrils, oral mucosa, and occasionally the limbs and udders (108). Although mortality is usually low, the disease has substantial welfare implications. Lesions are often painful and ulcerative, interfering with feeding, suckling, and normal social interactions. Outbreak reports from Kenya and Sudan show 100% morbidity in affected calf groups, with inability to suckle properly and frequent secondary infection; CCE contributed to “high calf mortality” and debility (109, 110). In Ethiopia and Sudan, herd-level mortality of 6–9% has been documented, almost entirely in young animals (111, 112). Young camels are particularly vulnerable, as oral lesions may lead to reduced milk intake, dehydration, and secondary infections. In adult animals, udder lesions can cause discomfort during milking and nursing (113). Camel Contagious Ecthyma is also zoonotic; human infections from camels have been documented, influencing how affected animals are handled and sometimes prompting rough or fear-based management (114).

### 4.6 Peste des petits ruminants

Natural outbreaks in Sudan, Iran and Kenya show that dromedary camels can develop overt PPR with fever, nasal–ocular discharge, oral lesions, diarrhea, respiratory distress, weakness and sudden death. Mortality in Sudanese outbreaks averaged 7.4% (range 0%−50%), disproportionately affecting pregnant and recently calved she-camels and adults (115). Iranian cases also showed severe dehydration, pneumonia, dermatitis, keratoconjunctivitis and multi-organ lesions (116). In Kenyan “camel sudden death syndrome,” affected camels were anorexic, emaciated, febrile and too weak to keep up with the herd, with diarrhea and ocular–nasal discharge preceding death (117). Experimental infection with a virulent strain produced seroconversion without clinical signs in one study (118), while transmission experiments showed infected camels can pass PPRV to sheep and goats despite only mild disease themselves (119).

The disease is particularly relevant in mixed species grazing systems, where camels share environments with infected sheep and goats. Welfare impacts may be compounded during outbreaks by movement restrictions, loss of access to grazing, and limited veterinary care. As PPR eradication programs expand, understanding the welfare consequences of both infection and control measures in camels becomes increasingly important (120).

### 4.7 Bovine viral diarrhea

Serological surveys show that dromedary camels are commonly exposed to BVDV, with antibody prevalences from ~1%−5% in Ethiopia, Egypt and Iran up to 16%−23% in Sudan, Tunisia and Turkey, and >40% in some Egyptian and Algerian populations. Many sampled animals were apparently healthy, confirming that infection is frequently subclinical Serological surveys show that dromedary camels are commonly exposed to BVDV, with antibody prevalences from ~1%−5% in Ethiopia, Egypt and Iran up to 16–23% in Sudan, Tunisia and Turkey, and >40% in some Egyptian and Algerian populations (121–127). While many infections in camels appear subclinical, BVDV has the potential to cause immunosuppression, reproductive disturbances, and poor growth. From a welfare perspective, subclinical infection is not benign; immunosuppressed camels may be more susceptible to secondary bacterial infections, leading to prolonged illness and reduced resilience (128, 129). Reproductive impacts, including early embryonic loss, can also indirectly affect welfare by contributing to repeated breeding, nutritional stress, and management interventions. The largely subclinical nature of BVDV infection makes welfare impairment difficult to detect in camels (130).

Table 3 presents an overview of the major viral infections affecting dromedary camels, outlining their prevalence, principal clinical features, transmission pathways, and public health significance. This synthesis serves as a practical reference for researchers and policymakers to support prioritization of surveillance efforts, vaccination programs, and biosecurity strategies.

**Table 3:** Viral diseases of dromedary camels: welfare impacts, mortality, and zoonotic concerns.

Disease	Virus	Typical clinical severity in camels	Direct welfare impact	Indirect welfare impact (management/policy)	Zoonotic risk
MERS	MERS-CoV	Mild or subclinical	Low at individual level	Trade bans, culling, neglect, stigma	Very high
Camel pox	Camel pox virus	Moderate–severe	Painful skin lesions, anorexia	Exclusion from work/markets	Low
RVF	RVF virus	Moderate–severe	Fever, abortion, neonatal mortality	Movement bans, emergency slaughter	Very high
Rabies	Lyssavirus	Severe, fatal	Neurological distress, fear	Rapid euthanasia, isolation	Very high
Contagious ecthyma	Parapoxvirus	Moderate	Painful oral/skin lesions	Reduced feeding, handling avoidance	Moderate
Peste des petits ruminants	Morbillivirus	Variable	Respiratory, enteric distress	Grazing restrictions	Low
Bovine viral diarrhea	Pestivirus	Mostly subclinical	Immunosuppression	Secondary infections	Low

## 5 Pathophysiological and behavioral indicators of welfare impairment

### 5.1 Physiological stress indicators

Disease-associated welfare compromise in camels is reflected in measurable physiological changes, particularly in stress hormones, acute-phase proteins, oxidative stress markers, and hematological profiles. For example, serum cortisol increases markedly in diseased animals, rising from approximately 10.3 ng/ml in healthy camels to 19.4 ng/ml in surra and exceeding 100 ng/ml in metritis cases, indicating severe stress (131, 132). Similarly, acute-phase proteins such as haptoglobin (~1.6 g/L), serum amyloid A (~26 mg/L), and fibrinogen (~5.1 g/L) are significantly elevated in respiratory and systemic infections (133). Oxidative stress markers, including malondialdehyde, are increased alongside reductions in antioxidant enzymes e.g., superoxide dismutase (3.1 ± 0.22 U/mg Hb), catalase (10.4 ± 1.3 U/mg Hb), glutathione (4.42 ± 0.65 mmol/g Hb), reflecting ongoing inflammatory damage (134). These changes are often accompanied by leukocytosis, neutrophilia, anemia, and altered biochemical profiles. Together, these biomarkers provide objective, quantifiable evidence linking infectious disease to compromised physiological welfare in camels.

### 5.2 Behavioral indicators

Behavioral changes are often the earliest and most practical field indicators of compromised welfare in camels. Common signs across bacterial and viral diseases include; Lethargy and reduced activity, Diminished grooming and coat care, Decreased feed and water intake, social withdrawal, reduced participation in group movements or resting apart from the herd, altered posture (e.g., reluctance to rise, abnormal leg positioning due to pain) and changes in gait and Changes in vocalizations—groaning, increased grumbling or silence in typically vocal individuals (13, 16). In working or racing camels, loss of willingness to move, balking during loading and reduced responsiveness to cues are important welfare indicators that may signal underlying disease rather than “stubbornness” (135).

Camel welfare indicators reported in field studies show substantial levels of compromise across production systems. In market camels in Qatar (*n* = 132), 62.9% had low body condition, 64.1% showed high thirst scores, and 38.6% displayed visible clinical disease signs, while hobbles and tethering were recorded in 20.5 and 3.8% of animals, respectively (16). In pastoral camels in Pakistan (*n* = 510), common problems included skin disorders (35.5%), discharges (27.5%), open wounds (10.0%), and pain-inducing procedures such as branding or nose pegging (29.8%) (136). Among working camels in Ethiopia (*n* = 384), 52.3% had wounds, 32.3% were in poor body condition, and 53.4% were tick infested (137). Experimental transport studies further showed elevated cortisol, neutrophilia, dehydration, reduced feeding, and abnormal resting behavior, confirming significant physiological stress during movement. Collectively, these data provide direct quantitative evidence that pain, injury, thirst, disease, and management-related distress are common welfare concerns in camels.

Table 4 summarizes current and potential strategies for controlling infectious diseases in dromedary camels, including prevention, diagnosis, treatment, and key research gaps. Together, these elements provide a practical framework to inform policy development, resource allocation, and future research priorities.

**Table 4:** Key physiological and behavioral indicators for assessing welfare impairment in dromedary camels with infectious diseases.

Welfare domain	Indicator type	Specific indicator	Associated disease states	Field applicability
Health	Physiological	Elevated cortisol, haptoglobin, SAA	Bacterial & viral infections	Moderate (lab-based)
Health	Clinical	Fever, dyspnea, diarrhea	Acute infections	High
Nutrition	Behavioral	Reduced feed and water intake	Most diseases	High
Behavior	Behavioral	Social withdrawal, reduced grooming	Chronic infections	High
Behavior	Locomotor	Lameness, reluctance to rise	Mastitis, abscesses	High
Mental state	Behavioral	Apathy, abnormal vocalization	Painful conditions	Moderate
Pain	Composite	Posture, facial tension, response to palpation	Mastitis, abscesses, tetanus	Emerging
Maternal behavior	Behavioral	Reduced nursing, calf rejection	Mastitis, brucellosis, RVF	High

### 5.3 Pain and discomfort assessment

Assessing pain in camels is challenging due to their stoic nature and the limited development of validated pain scales compared with horses or farm animals (138, 139). Nonetheless, emerging work on camel welfare suggests potential use of composite pain scores integrating posture, facial expression, locomotion, appetite and response to palpation. Facial expression scoring (e.g., a camel “grimace scale”) holds promise, mirroring developments in other species, but requires systematic validation (140). Owner and handler observations can also be valuable, although they may be biased by cultural norms that normalize certain signs of suffering. Training pastoralists and farm workers to identify subtle behavioral and facial indicators of pain is therefore a key component of welfare-oriented disease management (141, 142).

## 6 Socioeconomic and ethical dimensions

### 6.1 Impacts on livelihoods

In many dryland communities, camels are high-value assets; disease-related losses can be devastating. Brucellosis and reproductive infections reduce fertility and calving rates; trypanosomosis and mastitis reduces milk yield and quality (143, 144); camel pox and RVF cause direct mortality, reduced draught performance and decreased market value; and trade restrictions linked to MERS-CoV or RVF outbreaks disrupt income streams (45). These economic pressures can drive welfare–economic trade-offs. Owners may delay seeking veterinary care, continue to work mildly lame or febrile animals, or prioritize immediate family needs over recommended culling or isolation measures. Welfare-friendly management strategies must therefore be realistic within resource-poor contexts and supported by financial or social safety nets (145).

### 6.2 Zoonotic and public health concerns

Camels host several important zoonoses, including brucellosis, TB, MERS-CoV, RVF, Q fever, rabies and others posing a risk to herders, abattoir workers, veterinarians, traders and consumers of raw milk (146, 147). Public health campaigns may inadvertently portray camels as “dangerous” animals, influencing attitudes and care practices. Fear of infection may lead to neglect, abandonment, rough handling, or rapid sale of diseased camels, while reluctance to cull infected animals—often driven by emotional attachment and economic dependence—can allow disease transmission to persist (24, 148). One Health strategies must therefore recognize and manage these opposing pressures, balancing human health protection with the ethical treatment of camel (149). Camel production systems are highly heterogeneous across regions. Intensive dairy operations in the Gulf States operate under markedly different management, biosecurity, and veterinary infrastructures compared to mobile pastoral systems in East Africa or mixed agro-pastoral systems in South Asia. These structural differences influence pathogen exposure, diagnostic access, vaccination feasibility, reporting practices, and owner decision-making. Accordingly, the welfare implications of infectious diseases and the practicality of control strategies must be interpreted within specific ecological, economic, and socio-cultural contexts rather than assumed to be uniform across camel-rearing regions.

### 6.3 Ethical and cultural issues

In many Islamic and pastoral societies, camels hold a unique cultural and religious significance, often seen as symbols of resilience and prestige. As a result, stigmatizing diseased animals can conflict with cultural reluctance to euthanize or cull these valued animals, especially when diagnostic certainty is limited (150). The One Welfare concept emphasizes that animal welfare, human wellbeing and environmental health are intertwined. Ethical decision-making around culling, movement restrictions and market closures should therefore consider both animal suffering and the psychosocial and economic consequences for owners. Participatory approaches that involve herders, religious leaders and veterinarians in designing disease control strategies can enhance acceptability and compliance while improving welfare outcomes (151, 152).

## 7 Prevention and control strategies with welfare perspectives

### 7.1 Biosecurity and vaccination

Biosecurity measures—quarantine of newly purchased animals, hygiene at watering and feeding sites, safe disposal of abortive materials and vector control—are foundational for disease prevention (153, 154). Regionally available vaccines for camels include live attenuated camel pox vaccines, brucellosis and RVF vaccines used strategically in high-risk areas, while some countries employ brucellosis vaccination in co-grazing small ruminants or cattle to indirectly protect camels (22).

Vaccination is a powerful welfare tool: by preventing painful and debilitating diseases (Camel Pox, MERS-CoV, brucellosis) (155, 156), it reduces the frequency and intensity of negative experiences and the need for emergency treatments. However, vaccine campaigns must be planned to minimize handling stress, ensure appropriate needle hygiene and avoid overcrowding at vaccination points (22).

### 7.2 Early detection and veterinary care

Early diagnosis and prompt treatment of bacterial infections (e.g., mastitis, suppurative conditions, metritis) reduce suffering and improve prognoses. Strengthening community-based surveillance, training para-veterinarians and equipping mobile clinics in pastoral areas can shorten the interval between onset of signs and intervention (157). Analgesia (NSAIDs, local anesthetics) and humane handling should be standard components of treatment protocols, including obstetric assistance, abscess lancing and surgical interventions. Education of veterinarians and animal health workers on pain management in camels is critical, as pain is often under-recognized or untreated (158).

### 7.3 Integrating welfare into disease control

Disease surveillance and control programs rarely incorporate systematic welfare indicators. Incorporating simple welfare measures—body condition scoring, lameness scoring, lesion counts, behavioral indicators—into routine field visits and outbreak investigations would provide a more complete picture of disease impact (136). Veterinarians and extension workers can apply structured welfare assessment checklists based on the Five Domains Model to guide decisions between treatment and humane euthanasia, while also informing policy discussions on acceptable management practices in camel dairies, racing operations, and trekking programs (10, 16).

### 7.4 One health and one welfare approaches

**Figure 2:**
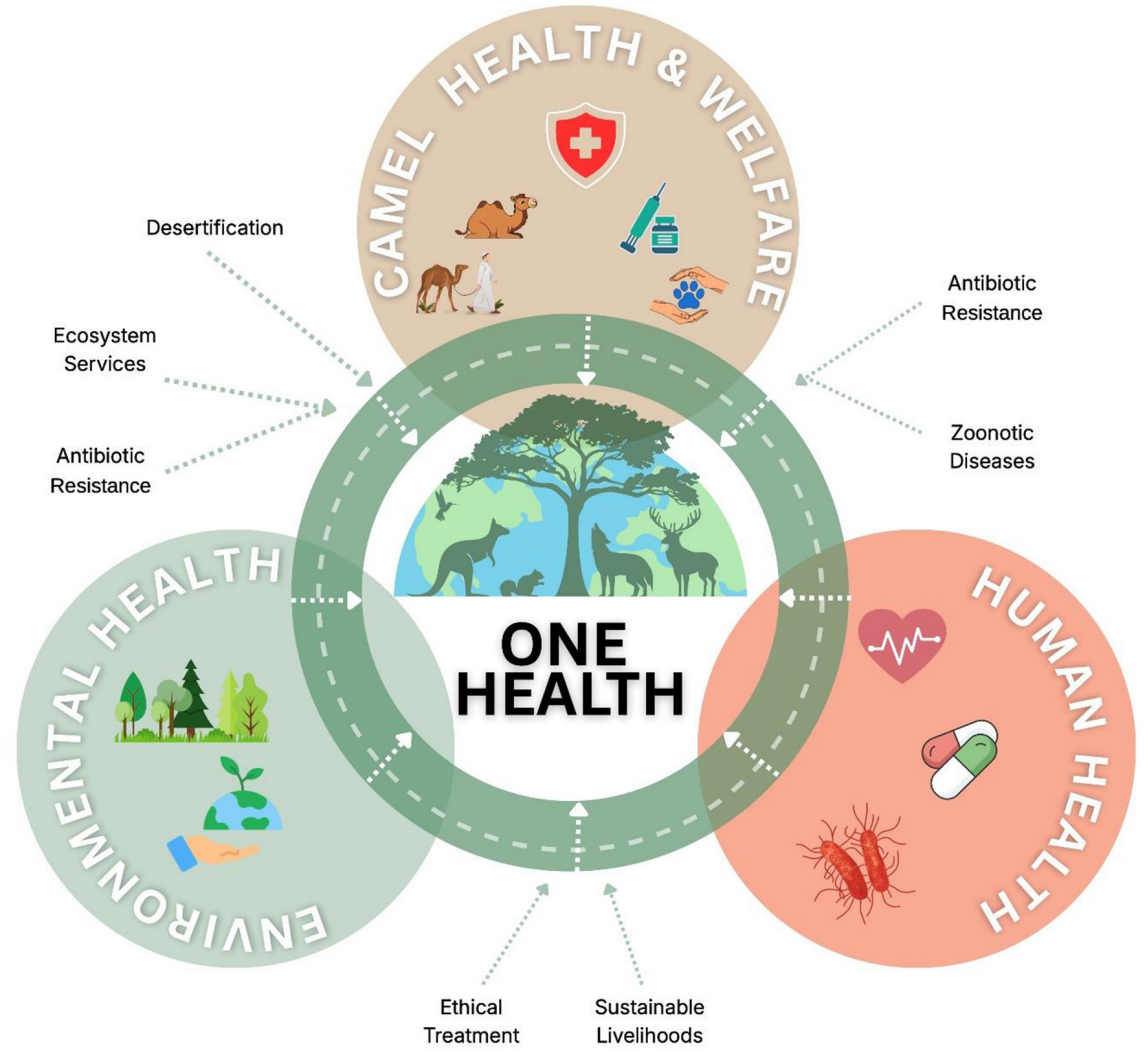
Integrating one health and one welfare approaches in camel husbandry practices.

The control of infectious diseases in dromedary camels is fundamentally aligned with the One Health framework, which emphasizes the interconnectedness of animal, human, and environmental health (Figure 2). Many camel diseases, including brucellosis, tuberculosis, MERS-CoV, and Rift Valley fever, are zoonotic and pose direct risks to pastoralists, veterinarians, and value-chain actors. Effective disease control strategies—such as vaccination, surveillance, and safe handling practices—therefore contribute not only to improved animal health but also to reduced human disease burden and enhanced food safety confidence (159). Middle East Respiratory Syndrome coronavirus mitigation requires harmonized surveillance across human and animal sectors, risk communication that avoids undue blame on camels and practical guidance for safe husbandry and slaughter (160, 161). Rift Valley Fever preparedness plans should combine vector control, vaccination and community training to reduce both animal mortality and human infections.

Building on this, the concept of One Welfare extends the One Health approach by explicitly incorporating animal welfare, human well-being, and socio-economic factors. In camel production systems, disease outbreaks and control measures (e.g., movement restrictions, culling, or trade bans) can influence livelihoods, human behavior, and the quality of animal care. For example, fear of zoonotic transmission may lead to neglect or harsh handling of infected animals, while economic dependency may discourage culling, thereby prolonging disease transmission and animal suffering. Integrating One Welfare principles ensures that disease control strategies are not only epidemiologically effective but also ethically responsible and socially acceptable, promoting sustainable and welfare-oriented camel production systems. Such integrated strategies support sustainable production systems and food security along with the resilience of dryland communities (Table 5).

**Table 5:** Welfare-oriented disease prevention and control strategies in dromedary camels placement.

Strategy	Target diseases	Welfare benefits	Potential welfare risks	Mitigation measures
Vaccination programs	Camel pox, rift valley fever, brucellosis	Reduces disease incidence, morbidity, pain, and mortality; prevents reproductive losses and chronic suffering	Stress during restraint; risk of injury from poor handling; transient post-vaccination reactions	Use calm, trained handlers; apply low-stress restraint techniques; schedule vaccination during cooler periods; monitor animals' post-vaccination
Movement control and quarantine	Rift Valley fever, brucellosis, camel pox, MERS-CoV	Limits disease spread; protects healthy animals and herds	Restricted access to grazing and water; risk of underfeeding, dehydration, and neglect; social stress	Ensure provision of adequate feed and water; limit duration of restrictions; monitor body condition and behavior regularly
Test-and-isolate strategies	Brucellosis, tuberculosis	Reduces exposure of healthy animals; enables targeted care of infected individuals	Social isolation stress; prolonged confinement; delayed treatment or euthanasia	Maintain visual and auditory contact with herd; provide enrichment; establish clear timelines for treatment or humane outcomes
Selective culling	Brucellosis, tuberculosis, severe RVF cases	Prevents prolonged suffering in advanced disease; reduces infection pressure in herd	Ethical concerns: poor handling or transport may cause distress; emotional impact on owners	Apply humane handling and slaughter standards; ensure informed consent and compensation schemes; veterinary oversight
Vector control	Rift Valley fever and other vector-borne infections	Reduces disease exposure and systemic illness; improves comfort by decreasing insect irritation	Potential toxicity from improper insecticide use; environmental impacts	Use approved products at recommended doses; integrate non-chemical methods (housing, drainage, repellents)
Improved hygiene and wound management	Mastitis, suppurative infections, skin and reproductive tract infections	Reduces pain, inflammation and secondary infections; promotes faster healing	Frequent handling may cause fear or stress; misuse of antiseptics	Train handlers in gentle techniques; minimize handling duration; use appropriate, animal-safe products
Enhanced veterinary surveillance and early diagnosis	All major bacterial and viral diseases	Enables early treatment; prevents progression to chronic pain and debility	Stress associated with repeated examinations or sampling	Use minimally invasive sampling where possible; combine clinical checks with routine husbandry
Biosecurity and herd management education	Zoonotic and non-zoonotic infectious diseases	Improves long-term health and welfare; empowers owners to recognize early signs of illness	Misinterpretation may lead to unnecessary isolation or avoidance of animals	Provide culturally sensitive training; emphasize welfare-positive practices and animal–human bonding
Pain management	Mastitis, abscesses, lameness, systemic infections	Alleviates suffering; improves recovery and behavioral comfort	Limited access to analgesics; improper dosing	Promote veterinary-guided analgesic use; include pain management in treatment protocols

### 7.5 Practical constraints and welfare trade-offs in disease control

Although vaccination, movement restrictions, quarantine and test-and-slaughter policies are commonly recommended for infectious disease control, their implementation in camel systems presents unique challenges. In mobile pastoral herds, repeated gathering for vaccination may induce handling stress and requires logistical coordination that is not always feasible. Cold-chain maintenance for vaccines can be unreliable in remote arid environments. Test-and-slaughter strategies, while epidemiologically effective in some livestock sectors, may be economically unsustainable for pastoral households, potentially discouraging disease reporting or incentivizing informal animal sales.

Movement bans and quarantine measures, although designed to limit pathogen spread, may inadvertently restrict access to pasture and water resources in dryland ecosystems, thereby introducing secondary nutritional and welfare stressors. These potential trade-offs highlight the need for context-sensitive disease control strategies that integrate epidemiological effectiveness with welfare and livelihood considerations.

## 8 Knowledge gaps and future directions

Despite growing recognition of camels' importance for food security and climate-resilient livestock systems, significant knowledge gaps remain at the intersection of infectious disease and animal welfare. Validated, widely applicable camel-specific welfare assessment tools are lacking, and existing frameworks, including those based on the Five Domains Model, require rigorous field validation across diverse production systems such as nomadic pastoralism, peri-urban dairies, tourism, and racing. Pain assessment tools, particularly behavioral and facial indicators, remain underdeveloped and underutilized. Epidemiologically, data on the prevalence, incidence, and burden of many bacterial and viral infections including chronic or subclinical conditions like tuberculosis, paratuberculosis, Q fever, and bovine viral diarrhea are limited, hindering effective risk prioritization and masking welfare impacts of prolonged illness. Longitudinal studies tracking herds over time are rare, limiting understanding of welfare trajectories during outbreaks or recovery.

The welfare consequences of disease control measures such as vaccination campaigns, test-and-slaughter, movement restrictions, quarantine, and culling are rarely evaluated, constraining holistic assessments of intervention effectiveness. Systematic evaluation of the welfare impacts and cost-effectiveness of disease control measures including vaccination campaigns, quarantine protocols, and culling strategies is needed, particularly in resource-limited and mobile production systems. Future research should prioritize developing practical, field-adapted welfare tools, linking infection dynamics with welfare outcomes through longitudinal studies, and integrating herder knowledge and socio-cultural considerations. Embedding animal welfare science within One Health and One Welfare frameworks is essential to ensure infectious disease control strategies protect camel wellbeing while supporting sustainable livelihoods and resilient dryland production systems. Addressing these gaps would enable policymakers to design disease management strategies that are evidence-based, economically feasible and welfare-informed.

## 9 Conclusions

Bacterial and viral infectious diseases impose substantial yet often underrecognized constraints on the welfare of dromedary camels, compromising all Five Domains through pain, fever, respiratory impairment, reproductive failure, behavioral disruption, and prolonged physiological stress, including in chronic or subclinical infections with hidden welfare costs. Welfare outcomes are shaped not only by the direct effects of disease but also by human responses driven by zoonotic risk, economic pressure, and control measures such as movement restrictions, quarantine, and culling, which can either alleviate or exacerbate suffering depending on their design and implementation. Welfare-oriented prevention and control strategies—particularly vaccination, early diagnosis, timely treatment, analgesia, and humane handling—offer clear benefits for camel wellbeing, public health, and pastoral livelihoods. However, major gaps remain, notably the lack of validated camel-specific welfare assessment tools, limited longitudinal data on welfare trajectories during disease and recovery, and poor integration of welfare indicators into surveillance programs. Embedding animal welfare science within One Health and One Welfare frameworks is therefore essential to support ethical, sustainable, and resilient camel health management systems as camel production expands under climate change.
